# Suicidal Ideation and Attempts Among Youth Living With HIV/AIDS Attending Botswana-Baylor Children’s Clinical Centre of Excellence: A Comparison Between Substance Users and Non-Substance Users

**DOI:** 10.1177/29768357251327567

**Published:** 2025-03-27

**Authors:** Leyla Baghirova-Busang, Anthony A Olashore, Mogomotsi Matshaba, Mooketsi Molefi

**Affiliations:** 1 Department of Family Medicine and Public Health, University of Botswana, Gaborone, Botswana; 2 Department of Psychiatry, Gaborone, University of Botswana, Botswana; 3 Botswana-Baylor Children’s Clinical Centre of Excellence, Gaborone, Botswana; 4 Department of Pediatrics, Baylor College of Medicine, Houston, TX, USA

**Keywords:** Youth, HIV/AIDS, substance use, suicidal ideation, suicide attempt, Botswana-Baylor Children’s Clinical Centre

## Abstract

**Introduction:**

Young people with HIV/AIDS (YLWHIV) who use substances early in life are at higher risk of suicidal thoughts and attempts. In Botswana, there is a lack of data needed to provide comprehensive care for this group. This study aimed to compare rates and predictors of suicidal ideation (SI) and attempts (SA) between substance users and non-users among YLWHIV at Botswana-Baylor Children’s Clinical Centre of Excellence (BBCCCE).

**Methods:**

This cross-sectional study, conducted from August 2022 to January 2023, involved 255 participants aged 15 to 24 and used the Youth Risk Behavior Surveillance System, the Oslo Social Support Scale, and the Patient Health Questionnaire-9. Chi-square tests were used to compare the prevalence of suicidal ideation and attempts among substance users and non-users, while logistic regression was used to identify factors associated with SI and SA.

**Results:**

SI was found in 29.4%, SA in 6%, and depression in 39.6% of the participants, while tobacco, alcohol, and illicit drug use were 20.4%, 45.9%, and 11.8%. Depression (adjusted odds ratios [AOR] 9.71, 95% CI: 4.23-22.33), being bullied (AOR = 5.42, 95% CI: 1.97-14.91), and family history of completed suicide (AOR = 6.56, 95% CI: 1.16-37.12) were associated with the outcome, SI. Self-reported good health status (AOR = 0.23, 95% CI: 0.05-0.98), lack of family support (AOR = 5.92, 95% CI: 1.45-24.10), and past-year tobacco use (AOR = 7.37, 95% CI: 1.18-46.10) were associated with SA.

**Conclusion:**

Due to the high rates of suicidal behavior among YLWHIV in BBCCCE, health facilities should scale up mental health services for this vulnerable group. This includes suicide risk assessments, bullying prevention, depression screenings, and counseling by trained lay health workers to promote their mental well-being.

## Introduction

The act of suicide, defined as the deliberate and self-inflicted cessation of one’s own life, is a prevalent global occurrence, transcending geographical boundaries.^1,2^ Annually, more than 700 000 people die by suicide, with a significantly greater number attempting to take their own lives.^1^ In 2019, the World Health Organization (WHO) identified suicide as the fourth leading cause of death globally among individuals aged 15 to 29 years.^3^ In Botswana, a country situated in Southern Africa, the age-standardized suicide rate is recorded at 20.2 per 100 000 population.^4^ This places the country in the 14th position out of 183 countries globally and seventh out of 54 African countries in terms of suicide rates.^4^ Suicidal behaviors, encompassing a spectrum of behavior from suicide attempts and preparatory behaviors like suicidal ideation, and planning, to completed suicide,^5^ pose a significant public health challenge on a global scale.^6^ Notably, a study conducted on suicidal behavior among undergraduate students at the University of Botswana found that 47.5% of respondents had experienced past-year suicidal thoughts, with 28.7% admitting to having attempted suicide during the past 12 months.^7^ Another study involving 201 young people living with HIV in Southwestern Nigeria reported a prevalence of current and lifetime suicidal ideation among the participants to be 14.9% and 33.3%, respectively.^8^

Individuals living with HIV/AIDS, particularly young people, are considered high-risk groups for experiencing thoughts of and engaging in suicidal behavior.^9,10^ During the adolescent stage of development, individuals are known to exhibit a propensity for engaging in higher levels of risk-taking behaviors. As youth progress from adolescence to adulthood, they are particularly vulnerable to involvement in perilous activities, including but not limited to substance abuse, violent behavior, suicidal ideation, and engaging in risky sexual practices.^11-13^ Youth living with HIV (YLWHIV) face the significant challenge of managing a stigmatized, sexually transmittable chronic illness; some in this population tend to turn to alcohol and illicit drugs as means of coping despite the detrimental impact on their quality of life and the increased risk of suicidal behavior.^14^ There is a paucity of studies exploring associations between suicidal behavior and substance use among HIV-infected youth in Botswana. These findings underscore the urgent need to prioritize mental health research and support, particularly for the vulnerable demographic of YLWHIV.

Substance use poses a significant risk in the YLWHIV as it increases the suicide risk along with other risk factors such as previous suicide attempts, pre-existing mental illness, and family history of suicide.^15^

The consumption of alcohol and illicit drugs is associated with various adverse effects. These substances are known to reduce inhibition, impair executive decision-making and judgment, and exacerbate impulse control issues. As a result, individuals under the influence of these substances are more likely to make poor decisions and have an increased propensity for attempting suicide.^16^ Chronic substance use may cause negative changes in behavioral, affective, and cognitive processes characterized as an underdeveloped regulation of aggression, sensation seeking, and impulsivity.^17^

Furthermore, the early debut of substance use, defined as first use prior to the age of 13 years, is associated with suicidal ideation and suicide attempts later in life, especially in adolescence, 13 years and older.^18-20^ A study conducted by Li et al involving 14 813 adolescents aged 10 to 19 from sub-Saharan African countries: Benin, Liberia, Mauritius, Mozambique, Namibia, Seychelles, and the United Republic of Tanzania found that the rate of attempted suicide was notably higher among early initiators (<13 years old) of tobacco use (30.03% vs 13.49%), alcohol (21.08% vs 12.77%), and illicit drugs (42.80% vs 13.97%) when compared to late initiators (⩾13 years old) and non-users.^21^ There is a strong association between marijuana use and an elevated risk of recurrent attempted suicide among students in the West, North, South, and East African regions.^22^ A correlation was found between suicidal behavior among homeless children and adolescents in Ghana and their history of tobacco use, past and present alcohol consumption, and marijuana use.^23^ Also, in South Africa, suicidal ideation and attempts were positively correlated with substance abuse among high school learners aged 13 to 23 years old.^24^

Limited research has been conducted on YLWHIV in African countries to investigate the connections between suicidal ideation/attempts and substance use.^25^ Existing studies primarily concentrate on Western and high-income nations such as the United States, Canada, Australia, and Europe,^26-28^ as well as on adolescents with unknown HIV status^17,21-23,29,30^ and adults living with HIV/AIDS.^31-34^ In Botswana, a country that ranks as one of the most heavily affected by HIV globally, the prevalence of HIV among young people, defined as individuals aged 15 to 24 years old, is 3.4%.^35^ Despite this high HIV prevalence, there is almost no exploration of the prevalence of suicidal behavior in this population. A recent study^36^ conducted among adolescents living with HIV aged 12 to 25 years and attending Botswana-Baylor Children’s Clinical Centre of Excellence (BBCCCE) revealed a prevalence of suicidal behavior at 18.8%. However, it is worth noting that this study utilized a non-probabilistic selection technique, which is prone to selection bias and may not fully capture or be representative of the said population,^37^ thus highlighting the need for a study with a better design that comprehensively covers this demographic. Another study in Addis Ababa, Ethiopia,^38^ involving HIV-infected youth aged 15 to 24 years, found that the magnitude of suicidal ideation and attempts were 27.1% and 16.9%; however, it did not make comparisons between drug users and non-drug users necessitating the current study.

In Botswana, mental illness is thought to be chronic and incurable, with mixed beliefs about the availability of treatment. This concept, coupled with the stigma and discrimination associated with both HIV and mental illness, as well as the shortage of mental health professionals in the country, limits the YLWHIV from getting the relevant services. In addition, co-morbid depression among HIV-positive children and youth is significantly associated with suicidal ideation and suicide attempts.^26,38,39^ Moreover, adolescents with depression and past-year experiences of violence (physical, sexual, or emotional abuse, as well as injuries inflicted with the intent to injure or kill) have a higher risk of suicidal ideation compared with their counterparts without such encounters.^40^ Conversely, some of the factors are known to be protective from suicidal behavior. Thus, physical activity, namely exercising 4 to 5 days a week, significantly reduces the adjusted odds of sadness, suicidal ideation, or suicide attempts in comparison with exercising 0 to 1 day a week among school-going students, improving their health status.^41^ Support from family and society is another protective factor for both suicidal ideation and attempts among people living with HIV.^42,43^

Meanwhile, the prevalence of suicidal ideation (51.5%) and attempts (40.1%)^7^ and substance use (past-year prevalence was 49.4%, that of the current use was 37.9%)^44^ among school-going adolescents and university students with unknown HIV status is very high in Botswana, and it is concerning. It is logical to assume that this issue will be even more pronounced among the exceptionally vulnerable population and young individuals who are living with HIV. Therefore, the primary objective of this study was to address this knowledge gap by quantifying and comparing the prevalence of suicidal ideation and attempts between substance users and non-users among the youth living with HIV/AIDS who seek services in Botswana-Baylor Children’s Clinical Centre of Excellence. Additionally, the study aimed to identify the factors associated with suicidal ideation and suicide attempts within these groups. The findings of this study were anticipated to provide valuable insights that will contribute to the development of targeted interventions aimed at preventing suicide among HIV-infected youth. Ultimately, it is hoped that this research will support the formulation of policy recommendations for the Botswana National Policy on Mental Health.

## Methods

### Study design and period

This cross-sectional study was conducted from August 2022 to January 2023 among YLWHIV attending the Botswana-Baylor Children’s Clinical Centre of Excellence (BBCCCE) in Gaborone.

### Study setting

The study was conducted at Botswana-Baylor Children’s Clinical Centre of Excellence, a public-private partnership between the Government of Botswana and the Baylor College of Medicine—Baylor International Pediatric AIDS Initiative (BIPAI), which was launched in June 2003. BBCCCE provides free-of-charge, state-of-the-art pediatric HIV, oncology, and blood disorder care, medical and surgical treatment, support to children, adolescents, and their families at the main clinic in Gaborone and through decentralized outreach services in 15 out of 27 districts of the country.^45^

Over 2400 clients, including 1484 individuals aged 15- to 24 years, attend the BBCCCE in Gaborone, the capital city of Botswana. Around 280 clients aged 15 to 24 years old attend the Centre every month. The Centre operates from 06:30 am to 4:30 pm 5 days a week. It is located on the Princess Marina Hospital (PMH) campus, a tertiary care referral hospital in Gaborone, Botswana.

### Study population

Youth living with HIV/AIDS aged 15 to 24 years who attended BBCCCE during the data collection period, from the first of August 2022 to the 15th of January 2023, constituted the study population. Those clients who refused to consent to the study or took part in the pilot testing of the study instrument (see Data collection sub-section) were excluded.

### Sample size determination and sampling technique

The sample size for the study was calculated using a formula comparing 2 proportions, considering an estimated prevalence of suicide attempts among substance users and non-users to be 28% and 12%, respectively,^46^ at a 95% confidence level, 90% power, the critical value of the Normal distribution at α/2 of 1.96, and the critical value of the Normal distribution at β of .84. The sample size constituted 127 participants per group (substance users and non-users) or, in total, 254 individuals. The sampling frame consisted of the individuals aged 15 to 24 years who were to attend the BBCCCE within the data collection period, 840 out of the total number of 1484 YLWHIV aged 15 to 24 years attending the Centre in Gaborone. Systematic random sampling was employed to select 254 individuals to be interviewed from the list of eligible prospective participants. The sampling interval of 3 was determined by dividing the expected number of clients attending the BBCCCE during 3 months of the initially estimated data collection period into the sample size (840/254). Prospective study participants selected from the list, or sampling frame, were approached and asked to participate in the study while exiting the BBCCCE after their follow-up visit. Sometimes, the participants from the list were not present at the Centre during the data collection process; in such cases and in cases of refusal to participate in the study, the next person at the Centre after their follow-up visit was approached.

### Measures

The data collection tool comprised 3 questionnaires/scales: the Youth Risk Behavior Surveillance System tool (YRBSS), the Oslo 3-item Social Support Scale (OSSS-3), and the 9-item Patient Health Questionnaire (PHQ-9).

#### Socio-demographic variables

The socio-demographic part of the questionnaire included questions about the respondent’s sex, age category, education, religion, employment, and employment status of the father and mother. Age was divided into 3 categories: 15 to 17, 18 to 21, and 22 to 24 years. Educational level was defined as having completed primary, attending, or having completed secondary or tertiary education. Religion was assessed as Christian, not religious or “Other,” the latter included African traditional or Islamic religion. Employment status was assessed as “Employed,” “Unemployed,” or “Self-employed.” The employment status of the father and mother was assessed as “Employed” or “Unemployed.”

#### Youth Risk Behavior Surveillance System tool

The Youth Risk Behavior Surveillance System tool was used to assess the health risk behaviors among the respondents.^47^ The authors used 4 out of 6 original categories of health-related behaviors from the YRBSS that contribute to the leading causes of death and disability among youth and adults. These include: (1) Behaviors that contribute to unintentional injuries and violence, (2) Alcohol and other drug use, (3) Tobacco use, and (4) Inadequate physical activity. Two of the original categories of health-related behaviors were not included in this study. Sexual behaviors related to unintended pregnancy and sexually transmitted diseases were not investigated in order to avoid overloading the respondents with an excessive number of sensitive questions. Unhealthy dietary behaviors are not considered to be an important risk factor for suicidality; hence, they were not investigated either.

Measures adapted from the YRBSS questionnaire included interpersonal violence, bullying, physical activity, health status, academic performance, tobacco, alcohol, and illicit drug use. Interpersonal violence victimization includes teen dating violence, sexual violence, and bullying during adolescence, which are associated with later revictimization, substance use, physical and mental health issues, and suicidal ideation.^48^ In this study, interpersonal violence was assessed as involvement in a physical fight in the past 12 months; being bullied in the past 12 months was assessed separately. Physical activity was assessed with the question, “On how many of the past 7 days did you exercise or participate in physical activity for at least 20 minutes. . .” with “0 days” being marked as “no” and “1-7 days” as “yes.” Health status was assessed with the question, “How do you describe your health in general?” with answers “excellent,” “very good,” and “good” grouped as “good,” while “fair” and “poor” were grouped as “poor.” Academic performance was assessed as “good” if the participant’s latest grades were mainly A, B, or C and as “poor” in the case of grades D and below. Substance users were defined as those consuming any amount of alcohol, tobacco, and/or illicit drugs that can be consumed, inhaled, injected, or otherwise absorbed into the body.^49^ Tobacco, alcohol, and illicit drug use was assessed both in the past 12 months and past 30 days.

The following measures were added under the YRBSS: mode of HIV acquisition, motivating and demotivating factors. Self-reported mode of HIV acquisition, perinatal or behavioral, was included in the questionnaire to explore a possible difference in the prevalence of and level of association with suicidal ideation and attempts among these groups. The mode of HIV acquisition was assessed with a question: “When did you become infected?” with the options of “When you were a baby” or “When you were a teenager.” Motivating and demotivating factors were included in the YRBSS questionnaire to explore additional risk and protective factors that may be associated with suicidal ideation and attempts among the YLWHIV. Motivating factors were assessed using the question “What would motivate you to live” with “Family support,” “Children,” “Employment,” and “Other” as possible responses, while demotivating factors were measured by the following responses: “Lack of family support,” “Discrimination,” “Poor academic performance”, and “Other” to the question “What would not motivate you to live.” At a later stage, motivating and demotivating factors were grouped into “Family support,” “Lack of family support,” and “Other” to avoid errors in the logistic regression models due to small numbers in some of the above-mentioned categories of these factors.

#### Oslo Social Support Scale

The social support variable was derived from the Oslo Social Support Scale. The OSSS-3 provides a brief measure of social functioning and is considered one of the best predictors of mental health.^50^ It measures the number of people the respondent feels close to, as well as interest and concern shown by others and ease of obtaining practical help.^51^ The sum score ranges from 3 to 14, with high values representing strong levels and low values—poor levels of social support: (a) score of 3 to 8 = poor support; (b) 9 to 11 = moderate support; and (c) 12 to 14 = strong support.^52^ In this study, the social support variable was dichotomized by assigning “0” to a “3-8” point of poor social support and “1” to a “9-14” point of moderate-to-strong social support. A systematic review and meta-analysis of the studies among HIV/AIDS patients by Weldesenbet et al was used as a reference: social support in those studies was measured using the Oslo 3-item social support scale, and individuals who were scored less than 9 regarded as having poor social support.^53^

#### Patient Health Questionnaire-9

PHQ-9 is a rapid and effective tool for detection as well as for monitoring the severity of depression.^54^ Depression is a common mental disorder that presents with loss of interest or pleasure, depressed mood, disturbed sleep or appetite, decreased energy, feelings of guilt or low self-worth, and poor concentration in the past 2 weeks.

Each item of PHQ-9 is scored on a scale of 0 to 3 (0 = not at all; 1 = several days; 2 = more than a week; 3 = nearly every day). The total score ranges from 0 to 27 (scores of 0-4 are classified as “no” to “minimal depression,” 5-9 as “mild depression”; 10-14 as “moderate depression”; 15-19 as “moderately severe depression”; ⩾20 as “severe depression”).^54^ Findings of the study by Mufson et al^55^ among young adults affected by HIV indicate that a PHQ-9 cut-score of 7 increased sensitivity from 47% to 76% (95% CI: 50, 93) in comparison with a PHQ-9 cut-score of ⩾10. Thus, to increase the sensitivity of the PHQ-9 scale and based on the literature review—a study by Gebremariam et al among the persons living with HIV/AIDS in Ethiopia,^9^ in the current study, the depression variable was dichotomized as follows: “0-4” were assessed as “no depression” and “5-27” as “depression.”

#### Outcome variables

There were 2 outcome variables in the study: suicidal ideation and suicide attempts. Suicidal ideation was measured as a categorical dichotomous variable with “yes” or “no” in response to the question of whether a participant ever seriously considered attempting suicide during the past 12 months. Suicide attempt was measured as a dichotomous variable derived from a multiple-choice question on how many times 1 actually attempted suicide during the past 12 months with a “yes” in case the participant reported 1 or more suicide attempts and a “no” if no suicide attempt was reported.

### Data collection

Four research assistants, undergraduate medical students, were trained on the data collection tool for face-to-face administration and equipped with the skills to establish rapport and build trust with the study participants for them to share very sensitive information on suicidal ideation/attempts and substance use. The questionnaire was pilot-tested among 20 YLWHIV attending the BBCCCE before the beginning of the study to assess the validity of the instrument, and no modification was deemed to be needed as a result. The structured questionnaires were prepared in both English and Setswana to allow the participants to choose a language they were more comfortable with. The questionnaires were administered electronically by uploading the respondents’ answers to Research Electronic Data Capture (REDCap) software. Individual debriefing sessions were conducted for all respondents on suicidal ideation and suicide attempts before and after participation in the study. Those respondents who have had previous or current suicidality, depression, or substance use issues were referred to clinical psychologists after the completion of the questionnaire.

### Data analysis

All the data obtained from the interviews via the REDCap online platform were imported into the Stata/SE 18.0 software. Missing data were treated by omitting those cases with the missing data and analyzing the remaining data. Continuous variables such as age were summarized as medians and interquartile range. Categorical variables are presented as proportions and expressed in absolute numbers and percentages. Prevalence rates of suicidal ideation and suicide attempts among youth living with HIV/AIDS who seek services at BBCCCE were estimated by dividing the number of cases by the group sample size times 100. A chi-squared test was used to compare the prevalence of suicidal ideation and suicide attempts between the 2 groups: substance users and non-users among the youth living with HIV/AIDS at BBCCCE.

A bivariate analysis was employed, using logistic regression, to examine a possible association between each of the independent variables (tobacco, alcohol, illicit drug use, etc.) and suicidal ideation and suicide attempts. Thereafter, those independent variables that were statistically significant at *P* < .25 were entered into multivariable analysis to avoid overfitting of the regression models. The cutoff of *P* < .25 is based on Bursac et al,^56^ suggesting that more traditional levels, such as .05, can fail in identifying variables known to be important for the multivariable analysis. However, substance use has been known from the literature to affect suicidal behavior; thus, variables related to substance use were included in the multivariable logistic regression models regardless of their statistical significance in the bivariate analysis. The model was adjusted for possible confounding, for example, age and sex, using adjusted odds ratios with 95% confidence intervals (95% CI).

### Ethical approval and informed consent

The research protocol was approved by the University of Botswana’s Institutional Review Board (UBR/RES/IRB/BIO/GRAD/151), Health Research & Development Committee of the Ministry of Health, Botswana (HPRD: 6/14/1), and the Botswana-Baylor Clinical Centre of Excellence Institutional Review Board (BBCO-IRB-2109-29). Written informed consent was obtained electronically before initiating research procedures from the parent/guardian of 15- to 17-year-olds and 18- to 24-year-olds who agreed to participate in the study. In addition, assent was obtained from 15- to 17-year-olds. Informed consent and assent forms were uploaded into the REDCap database, where a provision was made to obtain electronic signatures from parents/guardians of 15- to 17-year-olds, adolescents aged 15- to 17-year-old, and 18- to 24-year-old participants. The informed consent and assent forms were availed in both English and Setswana, the native language of Botswana.

## Results

### Demographic characteristics of the YLWHIV

The total number of participants enrolled in the study was 255. The median age of the respondents was 21 years, with an IQR of 19 to 22. YLWHIV aged 18 to 21 years old accounted for 47.5% of the respondents. Females constituted the slight majority, with 51.8% (n = 132) of the participants. Most respondents had secondary education and were unemployed, at 71.4% and 67.4%, respectively. Christians constituted 83.1% of the participants. Demographic characteristics of the participants are presented in Table 1.

**Table 1.** Demographic characteristics of 255 YLWHIV at Botswana-Baylor Centre.

Variables	Frequency (n)	Percentage (%)
Age (n = 255)		
15-17	39	15.3
18-21	121	47.5
22-24	95	37.2
Sex (n = 255)		
Male	123	48.2
Female	132	51.8
Education level (n = 255)		
Primary	3	1.2
Secondary	182	71.4
Tertiary	70	27.4
Academic performance (n = 252)		
Good (grades A, B and C)	166	65.9
Poor (grades D and below)	86	34.1
Religion (n = 255)		
Christian	212	83.1
Not religious	40	15.7
Other^a^	3	1.2
Employment status (n = 255)		
Employed	70	27.5
Unemployed	172	67.4
Self-employed	13	5.1
Employment status of the father (n = 201)		
Employed	118	58.7
Unemployed	83	41.3
Employment status of the mother (n = 219)		
Employed	119	54.3
Unemployed	100	45.7

a Traditional and Islamic religion.

### Clinical characteristics of 255 YLWHIV

There were only 19 (7.4%) participants who reported behavioral mode of HIV acquisition. The overall prevalence of depression was 39.6%, with females (n = 58, 22.7%) affected more than males (n = 43, 16.9%). Most (n = 181, 71%) of the study participants had moderate-strong social support. Most (n = 138, 54.1%) of the respondents chose family support as one of the motivating factors. Other motivating factors included financial freedom (n = 28, 33.3%), self-motivation (n = 26, 31.0%), children and family and friends, both at 8.3% (n = 7), health and religion, both at 4.8% (n = 4), helping others (n = 2, 2.4%), etc. The category of “Other” of the demotivating factors included not succeeding in life (n = 21, 32.8%), family and relationship issues (n = 16, 25%), health condition (n = 11, 17.2%), lack of finances (n = 7, 10.9%), lack of emotional support (n = 6, 9.4%) and other factors (see Table 2).

**Table 2.** Clinical features of 255 YLWHIV at Botswana-Baylor Centre.

Variables	Frequency (n)	Percentage (%)
Self-reported mode of HIV acquisition (n = 255)		
Perinatal	220	86.3
Behavioral	19	7.4
Not sure	16	6.3
Substance use (n = 255)		
Tobacco		
Yes	52	20.4
Alcohol		
Yes	117	45.9
Marijuana		
Yes	28	11.0
Other drugs^a^		
Yes	2	0.8
Physical fight (n = 255)		
Yes	21	8.2
No	234	91.8
Bullied (n = 255)		
Yes	31	12.2
No	224	87.8
Depression (n = 255)		
Present	101	39.6
Absent	154	60.4
Physical activity (n = 255)		
Yes	151	59.2
No	104	40.8
Sports teams (n = 255)		
No team	158	62.0
One or more teams	97	38.0
Social support (n = 255)		
Poor	74	29.0
Moderate-strong	181	71.0
Health status (n = 255)		
Poor	53	20.8
Good	202	79.2
Motivating factors (n = 255)		
Family support	138	54.1
Other^b^	117	45.9
De-motivating factors (n = 254)		
Lack of family support	92	36.2
Other^c^	162	63.8

a Cocaine and injectable drugs.

b Financial freedom, employment, self-motivation, children, family, and friends, health, religion, helping others.

c Not succeeding in life, family and relationship issues, health condition, lack of finances, poor academic performance, lack of emotional support.

**Figure 1.**
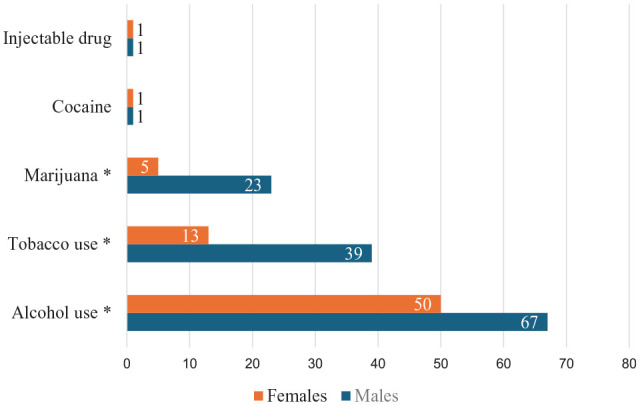
Pattern of substance use by gender among HIV-infected youth treated at Botswana-Baylor Centre. *Statistically significant variables at *P*-value < .01.

Prevalence of tobacco use, alcohol use, and illicit drug use among the respondents was at 20.4% (n = 52), 45.9% (n = 117), and 11.8% (n = 28), respectively. The prevalence of substance use differed between males and females, with males more likely to use tobacco (*P* < .01), alcohol (*P* < .01), and cannabis (*P* < .01; see Figure 1).

**Figure 2.**
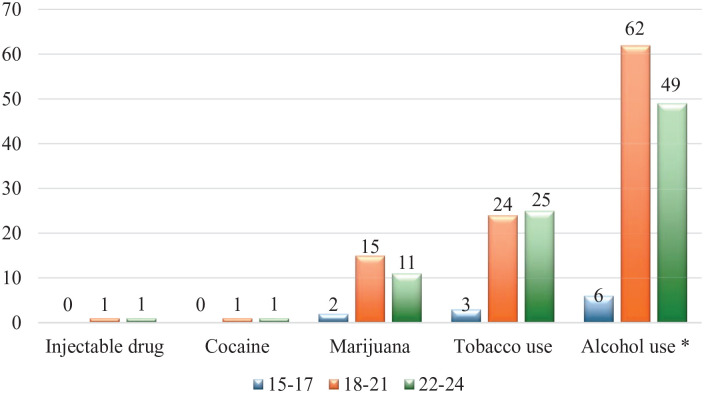
Pattern of substance use by age category among HIV-infected youth treated at Botswana-Baylor Centre. *Statistically significant variables at *P*-value ⩽ .01.

The highest proportion of tobacco use among all age categories was in 22- to 24-year-olds (n = 25, 48.1%), while the one of alcohol (n = 62, 53.0%) and cannabis (n = 15, 53.6%) use was in 18- to 21-year-olds. Those aged 18 to 21 years old were more likely to use alcohol (*P* < .01) compared to the older group of 22- to 24-year-olds (see Figure 2).

Out of 255 participants, 75 had suicidal ideation (SI). The overall prevalence of SI among the youth who attended the BBCCCE from the first of August 2022 to the 15^th^ of January 2023 was 29.4%. The overall prevalence of suicide attempts (SA) among the study participants was 6% (n = 15). An association did not emerge in the prevalence of suicidal ideation (SI) and suicidal attempts (SA) between 2 groups: substance users and non-users (see Supplemental Table 1). Statistically significant differences in the factors associated with suicidal ideation (SI) and suicide attempts (SA) between substance users and non-users are shown in the Logistic regression analysis of the factors associated with suicidal behavior section.

### Bivariate analysis of factors associated with suicidal behavior

In bivariate logistic regression analysis, family history of completed suicide, being involved in physical fights, being bullied, depression, and lack of family support were positively associated with suicidal ideation at a significance level of <.05. Good health status and moderate-strong social support were protective from developing suicidal ideation in the past 12 months (see Table 3).

**Table 3.** Factors associated with suicidal ideation in 255 YLWHIV at Botswana-Baylor Centre.

Independent variables	Suicidal ideation		COR (95% CI)	*P*-value	AOR (95% CI)	*P*-value
	(n)	(%)				
Sex (n = 255)						
Females	44	17.3	Ref		Ref	
Males	31	12.2	0.67 (0.39-1.16)	.156	1.11 (0.50-2.45)	.795
Family history of completed suicide (n = 255)						
No	69	27.1	Ref		Ref	
Yes	6	2.4	5.13 (1.25-21.09)	.023	6.56 (1.16-37.12)	.033
Employment status of the mother (n = 219)						
Employed	31	14.2	Ref		Ref	
Unemployed	35	16.0	1.53 (0.86-2.73)	.152	0.58 (0.27-1.25)	.163
Physical fight (n = 255)						
No	63	24.7	Ref		Ref	
Yes	12	4.7	3.62 (1.45-9.00)	.006	4.58 (0.98-21.49)	.053
Bullied (n = 255)						
No	55	21.6	Ref		Ref	
Yes	20	7.8	5.59 (2.52-12.39)	<.01	5.42 (1.97-14.91)	<.01
Depression (n = 255)						
Absent	20	7.8	Ref		Ref	
Present	55	21.6	8.01 (4.35-14.77)	<.01	9.71 (4.23-22.33)	<.01
Sports teams (n = 255)						
No team	52	20.4	Ref		Ref	
One or more teams	23	9.02	0.63 (0.36-1.12)	.119	0.53 (0.23-1.26)	.154
Health status (n = 255)						
Poor	50	19.6	Ref		Ref	
Good	25	9.8	0.37 (0.20-0.69)	.002	0.69 (0.27-1.75)	.438
Social support (n = 255)						
Poor	29	11.4	Ref		Ref	
Moderate-strong	46	18.0	0.53 (0.30-0.94)	.030	0.89 (0.39-2.06)	.792
De-motivating factors (n = 254)						
Other	37	14.6	Ref		Ref	
Lack of family support	37	14.6	2.27 (1.30-3.96)	.004	2.46 (1.00-5.55)	.060
Past year tobacco use (n = 255)						
No	61	23.9	Ref		Ref	
Yes	14	5.49	1.42 (0.69-2.92)	.366	0.71 (0.12-4.07)	.699
Past month tobacco use (n = 255)						
No	68	26.7	Ref		Ref	
Yes	7	2.75	0.87 (0.35-2.17)	.769	0.69 (0.08-6.04)	.740
Past year alcohol use (n = 255)						
No	43	16.9	Ref		Ref	
Yes	32	12.5	1.12 (0.65-1.93)	.693	0.85 (0.25-2.83)	.790
Past month alcohol use (n = 255)						
No	52	20.4	Ref		Ref	
Yes	23	9.02	1.06 (0.59-1.90)	.846	0.48 (0.13-1.75)	.268
Past month marijuana use (n = 255)						
No	70	27.5	Ref		Ref	
Yes	5	1.96	1 (0.34-2.94)	1.000	0.53 (0.07-4.28)	.554
Other illicit drug use^a^ (n = 255)						
No	73	28.6	Ref		Ref	
Yes	2	0.8	2.44 (0.34-17.64)	.377	3.03 (0.23-40.74)	.403

a Cocaine and injectable drugs.

Depression, past-year tobacco use, and lack of family support, among demotivating factors, were positively associated with suicide attempts at a significance level of <.05. Good health status was protective from attempting suicide in the past 12 months (see Table 4).

**Table 4.** Factors associated with suicide attempts in 255 YLWHIV at Botswana-Baylor Centre.

Independent variables	Suicide attempts		COR (95% CI)	*P*-value	AOR (95% CI)	*P*-value
	(n)	(%)				
Sex (n = 255)						
Females	11	4.3	Ref		Ref	
Males	4	1.6	0.37 (0.11-1.19)	.096	0.38 (0.09-1.71)	.209
Suicide attempt in family (n = 255)						
No	12	4.7	Ref		Ref	
Yes	3	1.2	3.08 (0.80-11.93)	.103	4.37 (0.48-39.74)	.191
Family history of completed suicide (n = 255)						
No	13	5.1	Ref		Ref	
Yes	2	0.8	5.12 (0.97-27.14)	.055	3.83 (0.27-54.25)	.320
Depression (n = 255)						
Absent	2	0.8	Ref		Ref	
Present	13	5.1	11.23 (2.48-50.91)	.002	5.69 (0.92-35.01)	.061
Sports teams (n = 255)						
No team	12	4.7	Ref		Ref	
One or more teams	3	1.2	0.39 (0.11-1.41)	.151	0.59 (0.09-3.91)	.588
Physical activity (n = 255)						
No	9	3.5	Ref		Ref	
Yes	6	2.4	0.44 (0.15-1.27)	.127	0.78 (0.17-3.70)	.759
Health status (n = 255)						
Poor	8	3.1	Ref		Ref	
Good	7	2.7	0.20 (0.07-0.59)	.003	0.23 (0.05-0.98)	.048
De-motivating factors (n = 254)						
Other	4	1.6	Ref		Ref	
Lack of family support	11	4.3	5.36 (1.66-17.38)	.005	5.92 (1.45-24.10)	.013
Past-year tobacco use (n = 255)						
No	9	3.5	Ref		Ref	
Yes	6	2.4	4.18 (1.40-12.52)	.011	7.37 (1.18-46.10)	.033
Past year alcohol use (n = 255)						
No	8	3.1	Ref	.634	Ref	
Yes	7	2.7	1.29 (0.45-3.67)		0.92 (0.13-6.69)	.934
Past month alcohol use (n = 255)						
No	11	4.3	Ref	.784	Ref	
Yes	4	1.6	0.85 (0.26-2.75)		0.51 (0.06-4.25)	.531
Past year marijuana use (n = 255)						
No	13	5.1	Ref	.465	Ref	
Yes	2	0.8	1.79 (0.38-8.52)		4.97 (0.25-97.25)	.291
Past month marijuana use (n = 255)						
No	14	5.5	Ref	1.000	Ref	
Yes	1	0.4	1 (0.12-8.09)		0.06 (0.001-3.03)	.162
Other illicit drug use^a^ (n = 255)						
No	15	5.9	Ref		Ref	
Yes	0	0	1		1	

a Cocaine and injectable drugs.

### Logistic regression analysis of the factors associated with suicidal behavior

The variables from the bivariate analysis at a statistical significance level of *P*-value < .25, along with the substance use variables, were included in the binary logistic regression models separately for suicidal ideation and attempts (see the crude and adjusted odds ratios in Tables 3 and 4). The following variables remained associated with suicidal ideation: family history of completed suicide (AOR 6.56, 95% CI 1.16-37.12) at a significance level of <.05, experiences of being bullied (AOR 5.42, 95% CI 1.97-14.91), and depression (AOR 9.71, 95% CI 4.23-22.33) at a significance level of <.01. While self-reported perinatal mode of HIV acquisition (AOR = 15.33, 95% CI 1.38-169.86) and past year marijuana use (AOR 31.30, 95% CI 1.02-962.91) also seemed associated with suicidal ideation, these variables were excluded from the models due to wide 95% CIs, which could have otherwise led to misinterpretation of the results. Thus, no significant association emerged between suicidal ideation and substance use in the multivariable logistic regression analysis.

Three factors were associated with suicide attempts in the multivariable analysis: self-reported good health status (AOR 0.23, 95% CI 0.05-0.98), lack of family support (AOR 5.92, 95% CI 1.45-24.10), and past-year tobacco use (AOR 7.37, 95% CI 1.18-46.10) at a significance level of <.05. No significant association emerged between suicide attempts and alcohol or marijuana use.

Age and sex were not found to be associated with SI (AOR 1.60, 95% CI 0.89-2.90; AOR 1.11, 95% CI 0.50-2.45) and SA (AOR 1.72, 95% CI 0.57-5.21; AOR 0.38, 95% CI 0.09-1.71) respectively.

## Discussion

The study sought to estimate and compare the prevalence of suicidal ideation and suicide attempts between substance users and non-users among YLWHIV, who seek services in BBCCCE and determine the associated factors. The overall prevalence of suicidal ideation in the study was 29.4%. This is higher than the pooled lifetime prevalence of suicidal ideation among young people with HIV/AIDS aged 9 to 25 years old and living in 7 Sub-Saharan African countries, USA, Thailand, and China, which was 24.4%.^57^ It is also much higher than suicidal ideation and self-injury reported by Brooks et al^36^ among 15% of youth from the BBCCCE in 2019, or 8.4% prevalence of suicidal thoughts reported by Casale et al among South African youth living with HIV/AIDS.^58^ The present investigation was conducted within the same demographic as the study conducted by Brooks et al. However, the latter was executed amidst the COVID-19 pandemic; this circumstance may account for their elevated proportion of YLWHIV with suicidal ideation, presumably influenced by the psychosocial implications associated with the pandemic.

The prevalence of suicidal ideation in our study, as determined by the Youth Risk Behavior Surveillance Survey and Patient Health Questionnaire, is lower than 33.3% reported among young people living with HIV in Nigeria.^8^ This disparity may be attributed to the older age of the participants in our study and the use of different assessment scales across the studies. While our study utilized the aforementioned assessment tools, the studies referenced used the MINI International Psychiatric Interview for Children and Adolescents Suicide questions (MINI-Kid). Nonetheless, the finding that Botswana’s suicidal ideation among YLWHIV was higher than the global average of 24.3%^57^ suggests the need to urgently implement strategies for addressing suicidal ideation in these individuals.

The overall prevalence of suicide attempts in this study was 6%, which is lower than the pooled estimate of suicide attempts among YLWHIV (13.05%) in a meta-analysis that included 14 studies^57^ but similar to a report from Thailand 7.9%, which was also among predominantly perinatally infected youth aged 18 to 25 years old.^59^ A much higher prevalence of suicide attempts, 24.2%, was reported by adolescents from poor urban settlements in Uganda.^60^ The observed variation may stem from cultural and socio-economic disparities within the study cohorts across different nations, as well as the use of varying assessment tools to screen participants for mental health disorders. However, a study by Olashore et al among adolescents from the same center reported 18.8% with an elevated risk of suicide,^61^ which further buttresses the need for suicide risk reduction interventions. The lack of specialized mental health service providers is widely acknowledged as a prevalent issue linked to the delivery of comprehensive mental health care services for YLWHIV, particularly in low-resource environments.^62^ Therefore, a task-shifting initiative such as providing basic mental health screening and counseling by trained lay health workers is recommended to compensate for the scarcity of specialist mental health care service providers in this health setting.^63^

No statistically significant association emerged between the prevalence of suicidal ideation/attempts in substance users and non-users, unlike in a previous study, which reported a higher prevalence of suicide attempts among alcohol and drug users compared to non-users, 57% versus 13%.^60^ The absence of a discernible relationship in the prevalence of suicidal ideation or attempts between the 2 groups of individuals may be attributed to the size and characteristics of our sample, although we hold the belief that these groups possess distinct characteristics and unique requirements. However, the current study found a significant relationship between suicide attempts and past-year tobacco use. It is known from the literature that HIV-infected individuals are at a higher risk of tobacco smoking^64^; however, the association between the latter and suicidal behavior in our study differs from other studies. For instance, a case-control study by Yu et al^65^ among 1395 HIV-negative men in China and another study by Gamarel et al^66^ among 2216 youth living with HIV aged 12 to 26 years from different US cities and Puerto Rico found that suicidal ideation, not suicide attempts, was associated with increased odds of reporting current tobacco use. This variation may be explained by differences in ages and other characteristics of the participants, larger sample sizes as well as different screening tools used by the study investigators. Consequently, we recommend conducting further research involving a meticulously selected, larger cohort and refining the selection of covariates with the aim of establishing meaningful associations between suicidal behavior and substance use. Such endeavors could prove instrumental in delivering personalized care services to young individuals living with HIV.

Our study findings provide insight into the prevalence of substance use among the youth attending the BBCCCE and associated factors necessitating counseling and smoking cessation initiatives among this population. Provision of social support to this demographic in the form of life skills training that promotes problem-solving, emotional regulation, and strong ties with family, friends, and a larger community may prove beneficial and protect these individuals from both substance use and suicide.^67^

The results of this study indicate that suicidal ideation is associated with depression, bullying, and a family history of completed suicide; these findings align with previous research conducted in diverse environments. In the study by Gebremariam et al,^9^ patients with depression were 2.5 times more likely to have suicidal ideation as compared to patients with no depression. Suicidal ideation was also associated with hopelessness and HIV-related stigma in Tanzania among adolescents living with HIV.^68^ In the same vein, depressive symptoms were positioned as a consistent risk factor for attempting suicide among adolescents with the perinatal mode of HIV infection,^10^ and it was also associated with moderate to high risk for attempted suicide in Uganda.^69^ This perhaps could be related to suicidal behavior, being an essential symptom of depression, according to the Diagnostic and Statistical Manual of Mental Disorders,^70^ and underscores the necessity of early and intensified depression screening, co-counseling for YLWHIV and comorbid mental health conditions, as well as the implementation of cognitive-behavioral, problem-solving, and pharmacological therapies.

As previously documented,^71^ bullying was positively associated with suicidal ideation in the present study. Bullying in any form is associated with internalized stigma, low self-esteem, and other internalizing symptoms such as depression, anxiety,^72^ and suicidality.^73^ In Malawi, YLWHIV has been reported to experience depression because of being bullied for taking medication, and the experience of being a victim has been associated with poorer outcomes across all mental health measures.^74^ Moreover, being bullied during childhood was significantly and positively associated with suicidal ideation later in life among HIV-positive men.^75^ The introduction of bullying prevention programs in the care of YLWHIV may reduce internalized stigma and promote mental well-being among them.

As reported in earlier texts, a family history of suicide correlated with suicidal ideation in our cohort.^9,76^ Our study, though unable to offer a scientific rationale for the genetic propensity toward suicidal ideation, supports a previous claim that individuals with a family history of suicide demonstrate increased vulnerability to suicidal thoughts.^77^ This suggests the importance of including inquiries about family history of suicide as a predictive factor in client clinical interviews. In the same vein, the loss of a family member has been associated with both suicidal ideation and attempts in several studies.^69,78^ The experience of parental loss due to AIDS-related complications during adolescence compounds the challenges faced by young people living with HIV. This is particularly pronounced when it occurs during a child’s formative years in a community such as Botswana, where most children living with HIV are raised in single-parent households.^61^ While social support may not be a direct mitigating factor within this particular cohort, these results indicate the necessity for social interventions, such as empowering families, providing financial aid, offering institutional placement, delivering mental health services, providing in-home and residential health services, ensuring supervision, furnishing educational resources, offering housing assistance, facilitating in-home care, promoting socialization, ensuring proper nutrition, and providing respite care.

Our study also established associations between suicide attempts and self-reported good health status as well as lack of family support. Self-reported good health status was protective against experiencing a suicide attempt. It, however, seems to be an isolated finding not corroborated by the existing literature, as the authors did not find any study with the association between these variables. Taking into consideration the high magnitude of the suicidal behavior and depression in our study, it is not surprising due to already compromised mental health and health in general. Lack of family support was another factor associated with suicide attempts in this study. Family support is an important part of social support that is linked to better mental health and a lower risk of suicide among people living with HIV/AIDS.^79,80^ Higher social support, especially from caregivers, who are mostly family members, was found to be a protective factor against mental health difficulties among adolescents living with HIV in Namibia.^81^ Conversely, negative interaction with the family, which included feeling disliked by family, feeling emotionally distant from family, or feeling exploited by family, was significantly associated with suicidal ideation among people living with HIV in Nepal.^42^ This finding necessitates counseling among HIV-infected youth and their families to improve family ties and acceptance.

Furthermore, early recognition and professional interventions can prevent some behavioral issues experienced by adolescents.^82^ These may include the implementation of peer group intervention,^83^ as this has significantly reduced depression, one of the major factors observed to increase the risk of suicide in this cohort.^84^ Also, given the high unemployment rate among HIV-infected youth, it is advisable to offer various community fundraising projects to provide them with meaningful occupation opportunities and combat stigma, discrimination, and social exclusion.

### Study limitations

One of the study’s limitations is that it is not possible to establish the temporal relationship between the predictors and the outcome variables—suicidal ideation and suicide attempts due to its cross-sectional nature. Another limitation is that this study is restricted to a specific group of young people living with HIV who attend the BBCCCE; therefore, its findings may not apply to the greater population of youth in Botswana. There may have been some selection bias in the sampling, as it is possible that young people with limited resources have less access to clinical services. Self-reporting on suicidal ideation/suicide attempts as an approach to measuring suicidality is also recognized as one of the limitations of the study. Moreover, some of the variables with very wide confidence intervals, like self-reported mode of HIV acquisition and past-year marijuana use, were removed from the multivariable logistic regression models despite having statistically significant associations with suicidal ideation or suicide attempts. The intention was to avoid having overparameterized models and reduce the chance of misinterpreting results.

In addition, it is important to consider the possibility that participants in the study may have provided responses that were influenced by societal expectations, potentially resulting in distorted findings. To address this concern, participants were specifically instructed on the significance of providing truthful responses and the potential impact of the data on the enhancement of the Botswana National Policy on Mental Health. It is crucial to also recognize another limitation of this study, namely the omission of an exploration of additional factors such as parental loss or emotional responses to HIV status. Finally, the outcomes of this study may be affected by the seasonality of suicidal behavior,^85^ and the findings might not be similar if the study is replicated during other seasons of the year.

## Conclusion

The prevalence of suicidal ideation among YLWHIV attending the Botswana-Baylor Clinical Centre of Excellence was high in our study. Depression, being bullied, and a family history of completed suicide are significant factors for suicidal ideation, while self-reported good health status, lack of family support, and past-year tobacco use are associated with suicide attempts among HIV-infected youth in BBCCE. Given the burden of mental health issues in the BBCCE, health facilities should scale up the provision of mental health services to this vulnerable population through the provision of suicide risk and depression screening as well as task-shifting initiatives such as counseling by trained lay health workers. The introduction of bullying prevention programs and the implementation of cognitive-behavioral and problem-solving therapies may promote the overall mental well-being of YLWHIV. Additionally, it is imperative to conduct more extensive research, including potential longitudinal studies, to gain a deeper understanding of the risk and protective factors, their impacts, and their interplay in relation to suicide behaviors.

## Supplemental Material

sj-docx-1-sat-10.1177_29768357251327567 – Supplemental material for Suicidal Ideation and Attempts Among Youth Living With HIV/AIDS Attending Botswana-Baylor Children’s Clinical Centre of Excellence: A Comparison Between Substance Users and Non-Substance Users Supplemental material, sj-docx-1-sat-10.1177_29768357251327567 for Suicidal Ideation and Attempts Among Youth Living With HIV/AIDS Attending Botswana-Baylor Children’s Clinical Centre of Excellence: A Comparison Between Substance Users and Non-Substance Users by Leyla Baghirova-Busang, Anthony A Olashore, Mogomotsi Matshaba and Mooketsi Molefi in Substance Use: Research and Treatment
