# Supplementary material for: Suicidal Ideation and Attempts Among Youth Living With HIV/AIDS Attending Botswana-Baylor Children’s Clinical Centre of Excellence: A Comparison Between Substance Users and Non-Substance Users
Source: Subst Use. 2025 Mar 27;19:29768357251327567. doi: 10.1177/29768357251327567 (PMC11951437; doi:10.1177/29768357251327567)
Supplement: sj-docx-1-sat-10.1177_29768357251327567 – Supplemental material for Suicidal Ideation and Attempts Among Youth Living With HIV/AIDS Attending Botswana-Baylor Children’s Clinical Centre of Excellence: A Comparison Between Substance Users and Non-Substance Users [file sj-docx-1-sat-10.1177_29768357251327567.docx]

**Supplementary materials**

Supplementary Table 1. Prevalence of suicidal ideation and suicide attempts between substance users and non-users among 255 YLWHIV at Botswana-Baylor Centre

| **Independent variable** | **Suicidal ideation %** | ***X^2^* value / Fisher’s exact test (SI)** | ***p-value* (SI)** | **Suicide attempts %** | ***X^2^* value / Fisher’s exact test (SA)** | ***p-value* (SA)** |
| --- | --- | --- | --- | --- | --- | --- |
| Alcohol users  Alcohol non-users | 30.0  28.9 | 0.0263 (75) | 0.871 | 5.9  5.8 | 0.0039 (75) | 0.950 |
| Marijuana users  Marijuana non-users | 32.1  29.1 | 0.1130 (75) | 0.737 | 10.7  5.3 |  | 0.219 |
| Other drug users  Other drug non-users | 50.0  29.1 |  | 0.583 | 0  6 |  | 1.000 |
| Tobacco users  Tobacco non-users | 34.6  28.1 | 0.8519 (75) | 0.356 | 11.5  4.4 | 3.7745 (75) | 0.052 |
